# Porcelain Crowns

**Published:** 1904-03-15

**Authors:** F. Ewing Roach


### PORCELAIN CROWNS.

BY .F. EWING ROACH, D.D.S.

The present-day porcelain crown properly constructed and judiciously applied is indeed a near approach to the ideal subsitute for the natural tooth. With the great variety of splendid bodies furnished by the dealers, including a vast selection of colors, a wide range of fusibility and most every other feature to be desired, there seems to be no limit to the possibilities of this department of our work.

The advent of the electric furnace has been the prime cause of latter-day rapid development in dental ceramics in general, and with the general development crown-work has not been overlooked. The electric furnace has brought porcelain work up from a place of drudgery and uncertainty to a really fascinating pastime. What wonder that so few undertake porcelain work when the old coke furnace was the only means of "baking."

Those of us who are to-day enjoying the luxuries of the electric furnace can best appreciate our advantages over the pioneers in the porcelain field by comparing our modern electric furnace and the old coke oven to our modern gas range and the old wood stove.

Nor has the rural dentist been forgotten in the advance. While not so desirable in some respects as the electric, the gas and gasoline furnaces afford adequate heat with such facility and reliability that the practitioner located in the most remote parts of the country may avail himself of the use of porcelain wherever its application is indicated and with results equal to those obtained by his city brother who uses the electric furnace.

Recognizing the inherent friability of porcelain and the exacting requirements in its manipulation, we cannot expect to obtain the best results if at any time we become careless or indifferent. A clear conception of the requirements and their strict observance, coupled with a judicious application, will insure results pre-eminently satisfactory in every respect. In determining when and where the porcelain crown is indicated, we must take into consideration primarily two things—possible stress and bulk of porcelain. For esthetic reasons the ten anterior teeth should, when possible, be crowned with porcelain and by reason of less masticatory stress being brought to bear upon the six anterior teeth they are especially favorable for this class of crown.

The porcelain crown is contra-indicated in inconspicuous places generally and always where a close bite precludes the employment of sufficient material for strength.

Aside from esthetic advantages the porcelain crown is decidedly more sanitary and possesses greater strength than the so-called Richmond crown with unprotected facing The attachment of the body built in on lingual surfaces supplements the pin attachment of facing—which does not obtain in the soldered facing—by uniting with and forming an integral part of the whole crown.

Without further discussion of its merits or demerits we will take up the essentials of construction and application of the porcelain crown from foundation.

First of all, let us see to it that our foundation is as it should be. Assuming that the pulp canals have been properly cared for, we proceed to prepare root for adjustment of band and dowel. Having ground off crown portion of tooth very nearly to the gum, the remaining enamel should be removed with cleavers, and the end of root so trimmed that it presents a cone-shaped appearance. The measurement should now be taken and band fitted. About 29 gauge platinum should be used and to insure a snug fit it is well to cut piece for band slightly shorter than measure, so that when fitting it may be forced to place and stretched a little if necessary. I have found it a good rule to make band slightly smaller and if necessary draw out some by a tap of the hammer on the anvil. This is easily done and insures an accurate fit; whereas, if the band were a little too large we are liable to let it go at that rather than cut out piece and solder.

If gold is used for soldering band a necessary precaution against subsequent opening of joint during baking is the lapped end joint. Before soldering the ends should be hammered or filed to a feather edge, thus avoiding unnecessary thickness where the lap occurs.

Having band accurately fitted to periphery of root and irregularity of free margin of gum so that there is no impingement upon peridental membrane it should be removed and root shortened—especially labially so that the free margin of gum will overlap and obscure from view the band, which is left exceedingly narrow at this point. Replace band on root and with a sharp pointed instrument scratch outline of end of root on inside of band, remove, trim to mark and readjust on root; after which the cap plate—30 gaugg platinum—may be soldered on. This is best done by placing band on piece to be used and soldering at some point of contact, after which the cap plate is easily burnished into contact with band and soldered without distorting same. Excess is trimmed off, cap is again adjusted to root and a sharp-pointed instrument is forced through cap plate into pulp canal—into which the post has been previously fitted—and the post forced through the small hole into the canal.

Impression and bite may now be taken, articulated model secured, facing ground in and waxed securely together with cap and post, preparatory to investment, after which facing and post may be soldered simultaneously thereby obviating the necessity of two investments. After soldering the piece should be boiled in sulphuric acid and all sharp angles removed from end of pins and post. The crown is now ready for the application of the porcelain. —*American Journal.*

